# Automatic Emergency Braking (AEB) System Impact on Fatality and Injury Reduction in China

**DOI:** 10.3390/ijerph17030917

**Published:** 2020-02-02

**Authors:** Hong Tan, Fuquan Zhao, Han Hao, Zongwei Liu, Amer Ahmad Amer, Hassan Babiker

**Affiliations:** 1 State Key Laboratory of Automotive Safety and Energy, Tsinghua University, Beijing 100084, China; th18@mails.tsinghua.edu.cn (H.T.); zhaofuquan@tsinghua.edu.cn (F.Z.); hao@tsinghua.edu.cn (H.H.); 2 Tsinghua Automotive Strategy Research Institute, Tsinghua University, Beijing 100084, China; 3 Research and Development Center, Saudi Aramco, Dhahran 31311, Saudi Arabia; AMER.AMER.4@ARAMCO.COM (A.A.A.); hassan.babiker@aramco.com (H.B.)

**Keywords:** intelligent transportation and traffic safety, automatic driving, fatality and injury, automatic emergency braking

## Abstract

The automatic emergency braking (AEB) system is an effective intelligent vehicle active safety system for avoiding certain types of collisions. This study develops a national-level safety impact evaluation model for this intelligent vehicle function, including the potential maximum impact and realistic impact. The evaluation model was firstly applied in China to provide insights into Chinese policymaking. Road traffic fatality and severe injury trends, the proportion of different collision types, the effectiveness of collision avoidance, and the AEB market penetration rates are considered in the potential maximum impact scenario. Furthermore, the AEB activation rate and the technology’s technical limitations, including its effectiveness in different weather, light, and speed conditions, are discussed in the realistic scenario. With a 100% market penetration rate, fatalities could be reduced by 13.2%, and injuries could be reduced by 9.1%. Based on China’s policy, the market penetration rate of intelligent vehicles with AEB is predicted to be 34.0% in 2025 and 60.3% in 2030. With this large market penetration rate increase of AEB, the reductions in fatalities and severe injuries are 903–2309 and 2025–5055 in 2025; and 1483–3789 and 3895–7835 in 2030, respectively. Considering AEB’s activation rate and its three main limitations, the adjusted realistic result is approximately 2/5 of the potential maximum result.

## 1. Introduction

The 2018 Global Status Report on Road Safety by the World Health Organization (WHO) highlights that the number of annual road traffic deaths has reached 1.35 million. The road traffic safety situation around the world is very serious, especially in developing countries [1]. In China in 2017, 63,772 people died and 209,645 were severely injured in road traffic accidents. Of these, 6111 deaths and 16,409 severe injuries were from rear-end collisions. In addition, 8558 deaths and 18,931 severe injuries resulted from pedestrian-single vehicle collisions [2].

The automatic emergency braking (AEB) system is considered to be an effective active safety system for avoiding rear-end and pedestrian collisions. This system is an advanced assistance system designed to identify imminent collisions and react by automatically activating the brakes, and is based on camera recognition of an object in front of the vehicle. Poisson regression was used to compare rates of police-reported crashes per insured vehicle year in the U.S. from 2010–2014 between passenger vehicle models with AEB and the same models without the optional systems, controlling for other factors affecting crash risk. The results showed that low-speed autonomous emergency braking (AEB) reduced front-to-rear crash rates by 43% and front-to-rear injury crash rates by 45% [3]. A methodology for quantifying the automatic emergency braking system’s effectiveness was proposed by Jeong. Using this methodology, based on Gyeonggi province crash data, AEB could prevent approximately 50% of the total rear-end crashes [4]. A meta-analysis was used to evaluate AEB’s effectiveness in different countries. The result showed a surprising 38% overall reduction in real-world, rear-end crashes for vehicles fitted with low speed AEB compared to a comparison sample of equivalent vehicles [5]. Hellman evaluated the crash mitigation effect of automated emergency braking systems based on data reported to insurance companies in Sweden. The result shows that rear-end frontal collisions were reduced by 27% for cars with AEB, compared to cars without the system [6]. Many existing studies have shown that AEB can reduce rear-end collisions by 25%–50%. A random parameters bivariate ordered probit model has been developed to examine factors affecting injury sustained by two drivers involved in the same rear-end crash [7]. Mixed logit models were developed to investigate the difference in driver-injury severity between single-vehicle and multi-vehicle accidents, in which the percentage of driver injuries in the event of truck brake defects, truck skidding/control loss, etc., were used as influential indicators [8]. Additionally, it can reduce pedestrian-single vehicle collisions. The changes in fatality and severe injury risks were quantified using risk curves derived by logistic regression of the German In-Depth Accident Study (GIDAS) accident database, queried for pedestrians hit by the front of cars from 1999 to 2007. For a 180° sensor view field, the effectiveness of preventing pedestrian fatalities and severe injuries from impact by the front of a car was 44% and 33%, respectively [9].

The total number of collisions that can be reduced by the AEB system in different countries is different due to the proportion of various types of traffic collisions in each country. In the United States, the Highway Loss Data Institute (HLDI) compared rates of insurance claims per insured vehicle year for Volvo S60 and XC60 vehicles with standard low-speed AEB with comparable vehicles without AEB. Volvos with low-speed AEB had 18% fewer collision claims, which covered damage to the at-fault driver’s vehicle [10]. In a similar study in the United Kingdom, Volvo XC60 models with standard low-speed AEB experienced 6% fewer own-damage claims [11]. Based on EU crash data, Wilmink reported that the AEB system can reduce injuries and fatalities by 7% each [12].

The safety benefits of AEB for a country are closely related to the market penetration rate of the active safety system. Jeong used VISSIM software to analyze the safety effects of autonomous driving for different market penetrations and proved the significance of the system [13]. Jeong asserted that considering the traffic conditions and market penetration rate is necessary to evaluate the effectiveness of active vehicle safety systems [14]. In the European Union, if the penetration rate of the AEB system was 11%, fatalities and injuries could be reduced by 0.9% and 1.3%, respectively [12].

This article evaluates the safety impacts of the AEB system in China. To predict the specific number of injuries and fatalities that can be avoided by AEB annually, we must consider AEB’s Chinese market penetration rate trend, Chinese policy regarding AEB systems, Chinese historical traffic collision data, and the proportion of different collision types.

This paper is organized as follows. The next section describes the research methods and data. Following that, predictions are made regarding injuries and fatalities without AEB in China through 2030, and the number of injuries and fatalities that could be avoided by AEB each year. The final section provides concluding remarks.

## 2. Methodology

**Figure 1:**
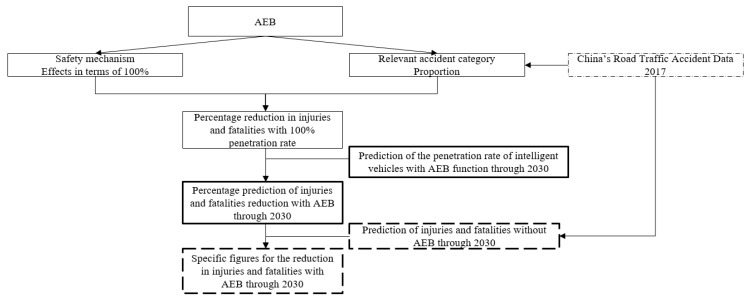
Evaluation model used in this study.

In this study, the numbers of injuries and fatalities without AEB through 2030 are multiplied by the percentage reduction in injuries and fatalities with AEB through 2030 to obtain the reduced numbers of injuries and fatalities with AEB through 2030, as shown in Figure 1. 

To get specific figures for the reduction in injuries and fatalities with AEB through 2030, China’s traffic accident trends, the proportion of various types of accidents, AEB technical details, AEB market penetration and other factors, such as weather and light distribution, must be considered, as shown in Equations (1) and (2). This is because the basis of AEB’s function is that the object in front can be recognized by the camera and radar. However, the camera and radar are less effective in bad weather and light conditions, such as sandstorms, fog, snow, and darkness.

(1) $${\mathrm{SI}}_{\mathrm{fat},y}={\mathrm{CN}}_{\mathrm{fat},y}*{\mathrm{EC}}_{\mathrm{fat}}*{\mathrm{PS}}_{\mathrm{fat}}*{\mathrm{PW}}_{\mathrm{fat}}*{\mathrm{PL}}_{\mathrm{fat}}*\mathrm{PO}*{\mathrm{PR}}_{y}$$

(2) $${\mathrm{SI}}_{\mathrm{inj},y}={\mathrm{CN}}_{\mathrm{inj},y}*{\mathrm{EC}}_{\mathrm{inj}}*{\mathrm{PS}}_{\mathrm{inj}}*{\mathrm{PW}}_{\mathrm{inj}}*{\mathrm{PL}}_{\mathrm{inj}}*\mathrm{PO}*{\mathrm{PR}}_{y}$$

where ${\mathrm{SI}}_{\mathrm{fat},y}$ is the specific number of fatalities reduced by AEB in year y; ${\mathrm{CN}}_{\mathrm{fat},y}$ is the predicted number of fatalities without the AEB system in year y; ${\mathrm{EC}}_{\mathrm{fat}}$ is the percentage of fatality reduction when all vehicles are equipped with the AEB system; ${\mathrm{PS}}_{\mathrm{fat}}$ is the proportion of driving speed less than 60 km/h for all fatalities in China; ${\mathrm{PW}}_{\mathrm{fat}}$ is the proportion of applicable weather conditions for all fatalities in China; ${\mathrm{PL}}_{\mathrm{fat}}$ is the proportion of applicable light conditions for all fatalities in China; PO is the proportion of users who turn on the AEB; ${\mathrm{PR}}_{y}$ is the AEB-equipped vehicle penetration rate to all vehicles in year y; and ‘inj’ indicates the injury data.

## 3. Data Description

### 3.1. Injuries and Fatalities without AEB through 2030

Traditional traffic accident prediction methods include the Smeed model, the Poisson random distribution model, the logistic curve model, and the back propagation neural network model [15,16]. These models have limitations, including large sample data and strong uncertainty, which lead to large deviations in prediction results. The Grey–Markov model is a mature model used to predict fatality and injury trends for road collisions in a country. The Grey–Markov model regards the occurrence of traffic accidents as a dynamic process, which can reveal the general trend of accidents over time and has the characteristics of high prediction accuracy and high reliability [15,16,17]. The key distinction of this model is that it uses the Markov theory to correct the Grey model prediction results for improved accuracy.

The injuries and fatalities caused by road traffic accidents in China during 2018-2030 was predicted based on data from 2008 to 2017 given by the Annual Report on Road Traffic Accidents of the People’s Republic of China [2]. The injury data in this article refer to severe injuries, because detailed data on severe injuries and fatalities are available, while similar data are not available for minor injuries. However, research on fatalities and severe injuries is meaningful. If detailed minor injury data are available in the future, they can be applied to the evaluation model in this study. The definition of several injury is physical disability, defacement, loss of hearing, loss of vision, loss of other organ functions, or other injuries that cause major harm to human health.

Based on historical accident data, the traffic accident fatalities without AEB are predicted to be 55,686 in 2020, 51,420 in 2025, and 47,484 in 2030, while the corresponding values for injuries are 188,285, 164,135, and 143,163, respectively. The maximum relative error of fatalities is −2.1% in 2013. The average relative error of fatalities is 1.0%. The maximum relative error of injuries is 2.7% in 2015. The average relative error of injuries is 1.3%.

### 3.2. Percentage Reduction in Injuries and Fatalities for AEB with Full Penetration

The evaluation method used here is the percentage reduction in injuries and fatalities with a 100% penetration rate multiplied by the AEB system penetration rate through 2030 to obtain the percentage of injury and fatality reduction with AEB through 2030.

The safety assessment framework for intelligent vehicle systems that cover three primary dimensions of exposure, risk, and consequence is a nine-point list of safety mechanisms, which was slightly adapted by Kulmala from the ten-point list proposed by Draskóczy [18,19,20,21]. 

In many cases, AEB will urgently brake the vehicle if it detects a danger of collision. The relevant AEB system safety mechanisms are described as mechanism 1 and mechanism 3 by Wilmink [12]. The definition of mechanism 1 is direct in-vehicle modification of the driving task and the definition of mechanism 3 is indirect modification of user behaviour. For mechanism 1, as mentioned in the introduction, the related collision types are pedestrian and obstacle collisions on a road, rear-end collisions, and head-on collisions. Cicchino reports that AEB reduces rear-end collision rates by 43% [3]. AEB has been shown to reduce front impact crash rates by 27% [22]. The effectiveness at preventing pedestrian fatalities and injuries due to impact by the front of a car was 44% and 33%, respectively [9], the same as collisions with road obstacles. For mechanism 3, behavioral compensation effects are assumed to be very limited, as AEB intervenes only in extremely dangerous situations and much later than normal driver braking [12]. Mechanism 3 will increase fatalities and injuries by 0.3% [21].

The percentage reduction in fatalities with a 100% AEB market penetration rate is calculated by Equations (3) and (4). The injury reduction percentage is similar.

(3) $${\mathrm{EC}}_{m1}=\underset{i}{\sum }EC_{i}*PC_{i}$$

(4) $${\mathrm{EC}}_{\mathrm{fat}}=\left(1+EC_{m1}\right)*\left(1+EC_{m3}\right)-1$$

where ${\mathrm{EC}}_{\mathrm{fat}}$ is the fatality reduction percentage for a 100% AEB market penetration rate; ${EC}_{i}$ is the percentage of fatalities reduced by AEB in a type *i* traffic collision; ${PC}_{i}$ is the proportion of fatalities caused by a type *i* collision in China; ${EC}_{m1}$ is an effect factor due to mechanism 1; and ${EC}_{m3}$ is an effect factor due to mechanism 3.

The proportion of fatalities and injuries caused by types of collisions in China is sorted from the latest Annual Report on Road Traffic Accidents of the People’s Republic of China (2017). The proportion of fatalities and injuries in the same type of collision is different, as shown in Table 1.

Based on the framework and updated proportions, the percentage reduction in fatalities is 13.22% when all vehicles are equipped with AEB, while the percentage reduction in injuries is 9.07%.

### 3.3. Penetration Rate of AEB in the Chinese Market

The AEB market penetration rate refers to the proportion of vehicles equipped with AEB functionality out of the total number of vehicles in China. The AEB new vehicle penetration rate refers to the proportion of new vehicles equipped with AEB functionality out of all new vehicles in a year. The AEB market penetration rate is calculated by Equation (5).

(5) $$PR_{y}=\frac{NPR_{y}*NV_{y}}{VO_{y}*Pop_{y}}$$

where ${PR}_{y}$ is the AEB penetration rate in year y; $NPR_{y}$ is the AEB new vehicle penetration rate in year y; ${NV}_{y}$ is the number of all new vehicles sold in China in year y; $VO_{y}$ is the number of vehicles owned per 1000 people in year y; and $Pop_{y}$ is the population of China in year y. 

The vehicle ownership per 1000 people through 2030 is calculated by Equation (6). The Gompertz function has been widely applied to estimate country-level vehicle ownership based on the economic factors [23].

(6) $${VO}_{y}=VOS*\exp(\partial \exp(\beta PGDP_{y}))$$

where $VO_{y}$ is the number of vehicles owned per 1000 people in China in year y; VOS is the saturation level of vehicle ownership per 1000 people in China; $\beta PGDP_{y}$ is the per capita GDP of China in year y; and α and β are two constants that vary with country.

The saturation level of vehicle ownership per 1000 people is 376 according to a prediction by the China Society of Automotive Engineers [24]. The GDP growth rates are predicted to be 6% in 2020, 3% in 2030, 2.4% in 2040, and 2% in 2050 [25]. The GDP growth rate through 2030 can be calculated by linear interpolation. China’s predicted population through to 2050 is from the United Nations Department of Economic and Social Affairs; it will reach an upper limit of 1.463 billion in 2030 [26]. The values of α and β are fitted to be −3.3546 and −0.00013 based on data for 2017 and 2018 [27], which are similar to the results for α and β calculated by Wu [23].

The new vehicle sales in each year through 2030 is calculated by Equation (7).

(7) $${NV}_{y}={VO}_{y}*Pop_{y}-\overset{t=18}{\underset{t=1}{\sum }}SR_{t}*NV_{y-t}$$

where $SR_{t}$ is the survival rate of the t-year after the vehicle is sold in China.

The survival rate of Chinese vehicles was assessed as 18 years [28]. The yearly sales volume of new cars from 2019 to 2050 is predicted based on the vehicle ownership in that year and the vehicle sales for the previous 18 years.

The data for the AEB new vehicle penetration rate were indirectly obtained by the new intelligent vehicle penetration rate, because AEB is a typical function of the lowest level of an intelligent vehicle, which is derived from the classification of intelligent vehicles in China [29]. According to national policy planning, 50% of new vehicles will be intelligent vehicles in 2020, 80% of new vehicles will be intelligent vehicles in 2025, and all new vehicles will be intelligent vehicles in 2030 [30]. The AEB new vehicle penetration rate was 0% in 2015. The AEB new vehicle penetration rate through 2030 is calculated by linear interpolation.

**Figure 2:**
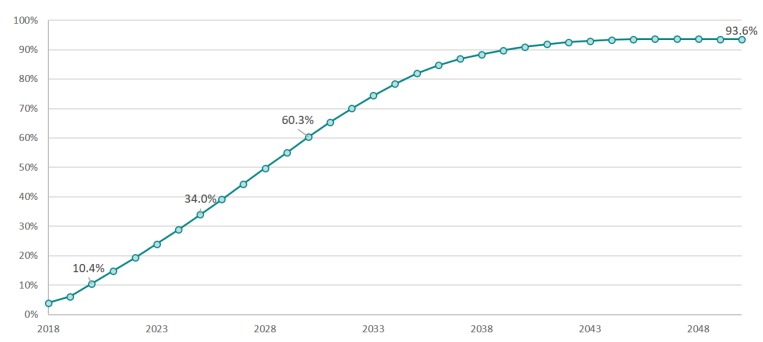
The automatic emergency braking (AEB) penetration rate through 2050 in China.

Based on the above data, the proportion of vehicles equipped with AEB out of total vehicle ownership through 2050 can be calculated to be 10.4% in 2020, 34.0% in 2025, and 60.3% in 2030, as shown in Figure 2.

### 3.4. AEB Key Safety Impact Influence Factors

There are some important limitations of the AEB system. First, the basis of AEB’s function is that the object in front can be recognized by the camera and radar. However, the camera and radar are less effective in bad weather and light conditions, such as sandstorms, fog, snow, and darkness. Second, AEB works efficiently only for speeds below 60 km/h. Tests of vehicles’ AEB function in China evaluate only whether the vehicle can brake safely at 40 km/h and 60 km/h [31]. Even at speeds of 40 km/h and 60 km/h, a large number of vehicle models equipped with AEB still have low test scores. The Cadillac XT4, Toyota Camry 2.5Q, Tesla Model S, BMW 330 Li, GAC GS8, and Chevrolet Malibu XL 535 scored 8.8, 8.1, 9.2, 6.2, 6.9, and 6.5 (10 is the best), respectively, on the AEB test conducted by i-Vista [32]. This outcome means that current AEB systems cannot fully guarantee safety at 60 km/h. When vehicle speed is above 60 km/h, AEB is ineffective with its current level of technology.

The gear distribution, light condition distribution, and weather distribution in collisions are extracted from the latest Annual Report on Road Traffic Accidents of the People’s Republic of China (2017), as shown in Table 2. The 2017 data are the latest available.

Notably, the original data only contains the gear information of the colliding vehicle. It is assumed that if the gear is below the fifth gear, the vehicle speed is below 60 km/h.

The proportion of speeds below 60 km/h in fatalities is calculated to be 63.01% by Equation (8).

(8) $${\mathrm{PS}}_{\mathrm{fat}}=\left(1.12\%+2.26\%+5.13\%+5.57\%+0.69\%\right)÷\left(1-19.09\%-41.02\%-16.44\%\right)=63.01\%$$

The proportion of speeds below 60 km/h in injuries is calculated to be 70.38% by Equation (9).

(9) $${\mathrm{PS}}_{\mathrm{inj}}=\left(1.41\%+3.06\%+6.21\%+4.83\%\right)÷\left(1-25.16\%-31.35\%-17.71\%\right)=70.38\%$$

Third, the driver may not always turn on the AEB system while driving. Delivering warnings to the driver earlier before braking will increase the time drivers have to avoid a potential collision. However, drivers who receive too many warnings that they deem unnecessary may grow to distrust the system and turn it off [33]. For 1000 drivers of vehicles from nine manufacturers, 93% drivers had forward collision warning or AEB turned on [34].

The proportions of good light conditions among fatalities and injuries are calculated to be 75.58% and 85.51%, respectively, except collisions that occur on roads without streetlights. It is assumed that AEB can only work effectively in collisions that occur in good weather conditions, including sunny and cloudy days. The proportion of good weather conditions among fatalities and injuries are calculated to be 88.36% and 88.82%, respectively.

Based on the above discussion, the probability that AEB is in good working condition is 39.14% for fatalities and 49.71% for injuries; these values were obtained by multiplying the quantized results of the limitations above. The assessment for each year’s reduction in fatalities and injuries is approximately 2/5 and 1/2 of the potential maximum results, respectively.

### 3.5. Scenario Definition

Based on the above discussion, three different scenarios are defined in this research to discuss AEB’s potential and realistic safety impact for reducing fatalities and injuries in China through 2030. Scenario 1 is a baseline scenario, representing injuries and fatalities without AEB through 2030. Scenario 2 is an optimistic scenario, indicating the potential maximum reduction in injuries and fatalities. In the optimistic scenario, it is assumed that an advancement in sensor technology enables AEB to work in bad weather and low light, that all drivers turn on the AEB system, and that AEB technology can effectively work over the entire speed range. Scenario 3 is a pessimistic scenario, representing realistic reductions in fatalities and injuries at the current technology level. The pessimistic scenario considers the AEB activation ratio and its technical limitations, including weather, light, and speed conditions.

## 4. Results and Discussion

**Figure 3:**
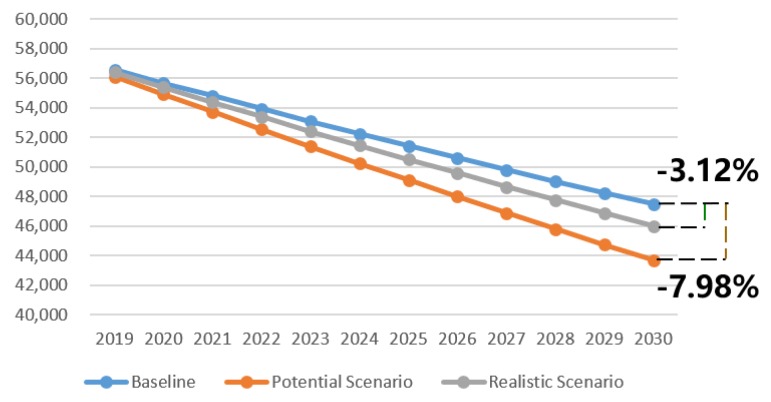
Road traffic fatalities for three scenarios through 2030.

**Figure 4:**
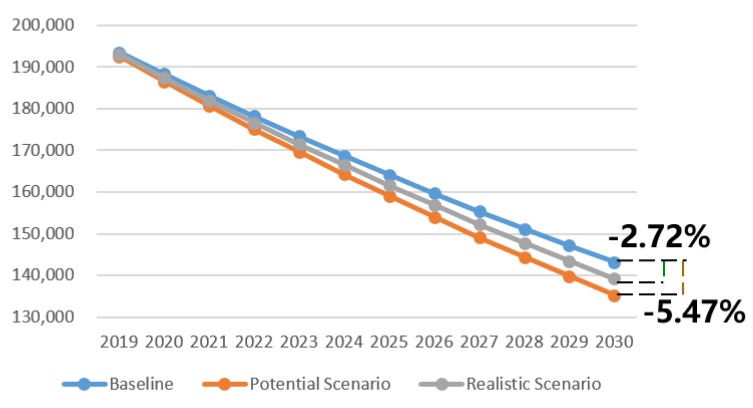
Road traffic injuries for three scenarios through 2030.

The specific reduction in fatalities and injuries with AEB each year was determined using the predicted number of injuries and fatalities without AEB, the percentage reduction for full penetration, and the market penetration rate of AEB each year. With a great increase in the market penetration rate, the reduction in injuries and fatalities will increase year over year. Based on the potential maximum result, 3789 fewer fatalities and 7835 fewer injuries will occur in 2030, and in 2025, there will be 2309 fewer fatalities and 5055 fewer injuries. Clearly, the AEB system can greatly improve road traffic safety in China. In 2030, the maximum potential percentage reduction in fatalities and injuries will be 7.98% and 5.47%, as shown in Figure 3 and Figure 4, because the proportion of vehicles equipped with AEB out of all vehicles in China is predicted to be 60.3% in 2030. If the government increases the AEB penetration rate through mandatory regulations or incentive policies, the fatality and injury reductions will be larger.

AEB technical limitations including speed, weather, and light conditions, as well as activation rates discussed in Section 3.4, are considered in the realistic pessimistic scenario. According to the realistic pessimistic result, 1483 fewer fatalities and 3895 fewer injuries will occur in 2030, while in 2025, 904 fewer fatalities and 2513 fewer injuries will occur. In 2030, the realistic percentage reduction in fatalities and injuries are 3.12% and 2.72%, as shown in Figure 3 and Figure 4. If sensor advances allow AEB to work effectively in bad weather and low light, the algorithm and execution advances enable effectiveness over the full speed range, and all drivers turn on the AEB; in addition, the annual reduction in fatalities and injuries would increase by 155% and 101%, respectively. 

In general, the result shows that the AEB system will reduce fatalities caused by road traffic collisions in China in 2030 by 3.12% to 7.98%, while the associated injury reduction ranges from 2.72% to 5.47%. In other words, the AEB system can avoid 1483~3789 fatalities and 3895~7835 injuries caused by road traffic collisions in China in 2030. 

**Figure 5:**
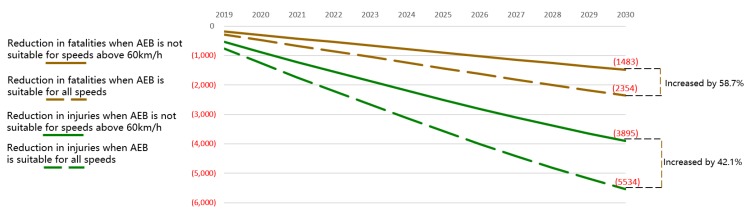
Reduction in fatalities and injuries in China with different speed suitability.

Of all the limiting factors, speed suitability provides the most room for improvement. Compared to the reduction in the realistic pessimistic scenario, the annual reduction in fatalities would increase by 58.7%, and the annual reduction in injuries would increase by 42.1% if AEB could work effectively over the full speed range, as shown in Figure 5. This outcome means that the reduction in fatalities would increase from 1483 to 2354 in 2030, and the reduction in injuries will increase from 3895 to 5534. Although full-range speed applicability can greatly improve safety, it is a comprehensive and technically difficult problem.

**Figure 6:**
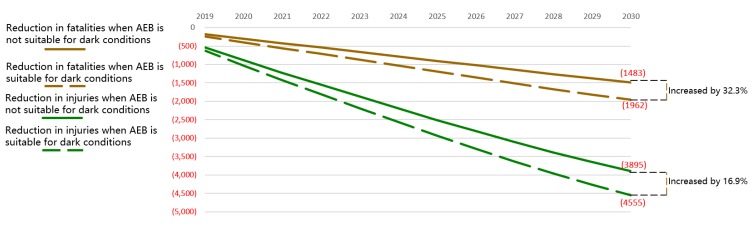
Reduction in fatalities and injuries in China with different light suitability.

The second-best way to improve AEB’s effect is to improve the light suitability of the sensor. In low light, cameras and radars cannot effectively identify objects and pedestrians ahead. Researchers should focus on the sensor’s low light suitability to address this challenge. Compared to the reduction in the realistic pessimistic scenario, the annual reduction in fatalities would increase by 32.3%, and the annual reduction in injuries would increase by 16.9% if AEB could work effectively in low light conditions, as shown in Figure 6. This outcome means that the reduction in fatalities will increase from 1483 to 1962 in 2030, and the reduction in injuries will increase from 3895 to 4555. 

**Figure 7:**
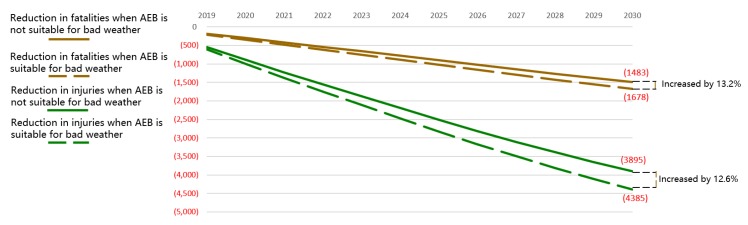
Reduction in fatalities and injuries in China with different weather suitability.

Improved effects from improved weather suitability follow the light suitability. In foggy weather, the sensor cannot recognize objects and pedestrians ahead. In snow, although the vehicle ahead can be identified, the braking system cannot work effectively. Compared to the reduction in the realistic pessimistic scenario, the annual reduction in fatalities would increase by 13.2%, and the annual reduction in injuries would increase by 12.6%, if AEB could work effectively in bad weather conditions, as shown in Figure 7. Weather suitability is a difficult challenge, involving sensors and execution.

**Figure 8:**
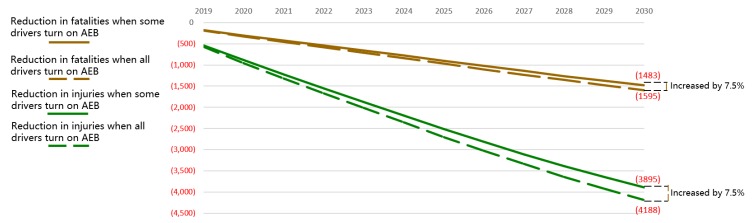
Reduction in fatalities and injuries in China for different AEB activation rates.

Activation rate has the smallest effect among all the limiting factors, because the current activation rate of AEB is high. Some manufacturers even set AEB to be on by default. Drivers generally acknowledge the necessity of warning and automatic braking. When all drivers are willing to activate the AEB functionality, the annual reduction in fatalities and injuries would increase by 7.5% compared with the realistic pessimistic scenario, as shown in Figure 8.

## 5. Conclusions

This is the first study to assess the national level safety benefits of AEB in China, taking into account the market penetration rate under China’s policy plan, and historical accident data in China. More innovatively, the effects of key factors including weather, light, speed, and activation rate were analyzed. The national model is applicable to evaluate the safety impact of other active functions equipped on intelligent vehicles, such as adaptive cruise systems, lane departure assist systems, etc. In addition, it can also be used to evaluate the national-level safety benefits of popularizing AEB technology in other countries.

The results clearly show that the AEB system can greatly improve road traffic safety in China. Compared to the predicted number of fatalities and injuries without AEB, the 2030 potential maximum percent reductions in fatalities and injuries in China are 7.98% and 5.47%, respectively, corresponding to the avoidance of 3789 fatalities and 7835 injuries. Technical limitations have a great impact on AEB’s safety benefits. Considering the system’s limitations, including weather, light, and speed conditions, and the activation rate, the realistic percentage reductions in fatalities and injuries are 3.12% and 2.72%, implying that 1483 fatalities and 3895 injuries could be avoided in 2030. Of all the limiting factors, the best path to improve AEB safety benefits is to improve its performance under a wider range of speed and light conditions.

Future work will focus on the safety impact of other active functions equipped on intelligent vehicles, such as adaptive cruise systems, lane departure assist systems, etc. After completing the safety benefit assessment of the main functions, the economic cost of accidents and casualties will be introduced to assess the economic benefits of intelligent vehicles from the perspective of reducing accident casualties.

## Figures and Tables

**Table 1:** The proportion and the number of injuries and fatalities in China’s collisions (2017).

Collision Type in 2017	Accidents	Proportion	Fatality	Proportion	Injury	Proportion
Pedestrian-single vehicle collision on a road	23,288	11.47%	8558	13.42%	18931	9.03%
Obstacle-single vehicle collision on a road	4179	2.06%	1904.5	2.99%	3765	1.80%
Single vehicle collision beside the road with pedestrian, obstacle or another single vehicle	35,015	17.24%	15,179.5	23.80%	31,175	14.87%
Head-on collision	14,495	7.14%	5064	7.94%	18,661	8.90%
Sideswipe collision	14,135	6.96%	3197	5.01%	15,497	7.39%
Angle collision	83,083	40.92%	18,955	29.72%	92,727	44.23%
Rear-end collision	15,481	7.62%	6111	9.58%	16,409	7.83%
Other collision with two vehicles	9904	4.88%	3492	5.48%	9033	4.31%
All other collision	3467	1.71%	861	1.35%	3456	1.65%
Total	203,049	100%	63,772	100%	209,645	100%

**Table 2:** The distribution of gear, light, and weather in China’s collisions in 2017.

Distribution	Category		Proportion of Fatalities	Proportion of Injuries
Speed distribution	Manual transmission	Neutral gear	16.44%	17.71%
		Gear 1	1.12%	1.41%
		Gear 2	2.26%	3.06%
		Gear 3	5.13%	6.21%
		Gear 4	5.57%	4.83%
		Gear >=5	8.67%	6.75%
		Reverse gear	0.69%	0.52%
		unclear	41.02%	34.35%
	Automatic transmission	Automatic transmission	19.09%	25.16%
Light condition distribution	Day		51.62%	61.00%
	Night with street-light		18.07%	20.20%
	Night without street-light		24.42%	14.49%
	Dusk		3.24%	1.78%
	Dawn		2.65%	2.53%
Weather condition distribution	Sunny day		73.98%	73.56%
	Cloudy day		14.38%	15.26%
	Rainy day		10.45%	10.34%
	Snowy day		0.46%	0.37%
	Foggy day		0.53%	0.36%
	Windy day		0.03%	0.03%
	Sandstorm		0.02%	0.02%
	Hail day		0.00%	0.00%
	Smoggy		0.02%	0.00%
	Other		0.13%	0.07%
